# Preparing to prescribe: How do clerkship students learn in the midst of complexity?

**DOI:** 10.1007/s10459-015-9606-0

**Published:** 2015-05-17

**Authors:** Lucy McLellan, Sarah Yardley, Ben Norris, Anique de Bruin, Mary P. Tully, Tim Dornan

**Affiliations:** Department of Educational Development and Research, Maastricht University, PO Box 616, 6200 MD Maastricht, The Netherlands; University Hospital of South Manchester, Southmoor Road, Wythenshawe, Manchester, M23 9LT UK; Camden, Islington ELiPSE, UCLH and HCA Palliative Care Team, Central and North West London NHS Foundation Trust, Unit D Well House, 23a Benwell Rd, London, N7 7BL UK; Royal Devon and Exeter NHS Foundation Trust, Barrack Road, Exeter, EX2 5DW UK; Manchester Pharmacy School, University of Manchester, Oxford Road, Manchester, M13 9PT UK; Centre for Medical Education, Queen’s University Belfast, Belfast, Northern Ireland BT7 1NN UK

**Keywords:** Medical students, Clinical competence, Prescriptions, Systems theory, Clinical medicine, Education

## Abstract

Prescribing tasks, which involve pharmacological knowledge, clinical decision-making and practical skill, take place within unpredictable social environments and involve interactions within and between endlessly changing health care teams. Despite this, curriculum designers commonly assume them to be simple to learn and perform. This research used mixed methods to explore how undergraduate medical students learn to prescribe in the ‘real world’. It was informed by cognitive psychology, sociocultural theory, and systems thinking. We found that learning to prescribe occurs as a dynamic series of socially negotiated interactions within and between individuals, communities and environments. As well as a thematic analysis, we developed a framework of three conceptual spaces in which learning opportunities for prescribing occur. This illustrates a complex systems view of prescribing education and defines three major system components: the “social space”, where the environmental conditions influence or bring about a learning experience; the “process space”, describing what happens during the learning experience; and the intra-personal “cognitive space”, where the learner may develop aspects of prescribing expertise. This conceptualisation broadens the scope of inquiry of prescribing education research by highlighting the complex interplay between individual and social dimensions of learning. This perspective is also likely to be relevant to students’ learning of other clinical competencies.

## Introduction

Prescribing forms an important part of day-to-day practice for doctors. Junior trainees regularly make decisions about whether, what and how to prescribe. This process entails combining knowledge learnt during pre-clinical pharmacology and physiology lectures with clinical assessment, judgement and skill. Teaching students to do this well is one of the most important challenges for modern day medical curricula, due to the potentially harmful effects of bad practice. Safe prescribing is a crucial part of patient care because even ‘household’ medications (such as ibuprofen and aspirin) and basic intravenous fluids can prove fatal if prescribed without recognition of a patient’s background and context. Furthermore, it is no exaggeration to say that prescribing practices contribute to one of the biggest worldwide health challenges: antimicrobial resistance. Improving the appropriateness and accuracy of future doctors’ prescriptions is a crucial part of enhancing patient safety at an individual level, health service efficiency at a regional and national level, and international welfare at a global level.

In 2009, the EQUIP study showed error rates of 8.4 and 10.3 % in prescriptions written by newly qualified doctors in their first and second years of clinical practice (Dornan et al. 2009). Errors occurred most commonly with antimicrobial drugs and incorrect dosage was the most frequent mistake. The reasons why errors occur were thought to be a “complex mixture of antecedent and contextual factors”; for example, miscommunication, lack of contextual and declarative knowledge and insufficient support in workplaces. In view of the complexity of working environments, errors were thought often to be a necessary adaptation to working conditions. In order to reduce error rates, the authors of that report suggested five target areas, four of which were educational innovations. An important issue was the transition from non-prescriber to prescriber, which is a major challenge for newly qualified doctors, and one they feel their undergraduate education does not prepare them for sufficiently (Morrow et al. 2012; Rothwell et al. 2012; Tallentire et al. 2011). Just as prescribing practice presents difficulties for newly qualified doctors, improving the quality of prescribing education is proving problematic for educators. Recent reviews found that interventions designed to improve undergraduate prescribing education are limited in their efficacy (McLellan et al. 2012; Ross and Loke 2009). This is likely to be due, at least in part, to prescribing often being taught as an isolated component of curricula, removed from the context of clinical care (McLellan et al. 2012). Theories of expertise development suggest that learning within authentic social contexts is fundamental to enabling learners to transfer what they have learnt into their clinical practice (Bereiter and Scardamalia 1986, 1993; Schmidt et al. 1990).

The importance of students interacting with real patients and having the opportunity to ‘learn by doing’ is well recognised (Bell et al. 2009; van der Zwet et al. 2011). This means that, if we are to prepare students to become confident, safe and efficient prescribers, then their training should include contextualised, whole-task practice of prescribing (McLellan et al. 2012; van Merriënboer and Kirschner 2007). Yet, with few exceptions (Sandilands et al. 2010; Smith et al. 2012), the published literature shows that students are rarely presented with educational encounters that afford them the opportunity to practice the whole task of prescribing: meeting a patient, identifying a need for drug therapy, appropriately selecting a drug, completing a prescription and being involved in the patient’s subsequent management. Instead, students learn about fragmented parts of prescribing, segregated from other aspects of medical practice. In removing the social context of prescribing and breaking it down into parts, the task is simplified. It is unsurprising, then, that medical students struggle to either reassemble the parts (a prerequisite to transfer) or recognise how to transfer this learning to the complex environments of medical practice.

Whilst suggesting that medical students’ formal prescribing education is overly simplistic in its approach, we acknowledge that a significant amount of learning takes place informally in workplaces and remains largely unevaluated. The extent to which students experience the realities of prescribing during their clinical placements and, more importantly, the conditions that support such prescribing education is currently unknown. Jörg et al. (2007) stated that “a profound understanding of pedagogical reality is necessary” in order to be able to improve this reality. So, before we make assumptions about how to improve the future of undergraduate prescribing education, we must explore its complex realities in the present.

This paper describes an exploratory research project investigating how senior medical students learn to prescribe, not only during tailored components of undergraduate curricula, but also as they engage with multiple different clinical teams and contexts (Holmboe et al. 2011). We were interested in students’ participation in prescribing-related encounters of any kind, whether organized or spontaneous, and how these experiences influenced students’ learning. Our primary research question was deliberately broad, in order to encompass the formal, informal and hidden curricula: how do final year medical students learn to prescribe? This paper describes a multi-method, multi-theory study, whose findings result from a convergence of different methods and perspectives. The differing paradigms that informed our work, outlined below, have in common an appreciation of the complexity of education, clinical practice and education research.

## Methods

Ethical approval for the study was granted by the University of Manchester ethics committee.

### Epistemology and theoretical orientation

Given the social nature of the environments in which prescribing takes place, it was logical to situate our work within a constructionist paradigm. This holds that perceptions of reality are mediated by social, cultural and interpersonal factors and that knowledge of phenomena is socially constructed (Crotty 1998). This perspective acknowledges that multiple accounts of reality exist and that meaning is generated mutually between researchers and participants, and also (in this research context) between different people involved in prescribing.

A number of different theoretical perspectives were relevant to our research question, including systems thinking (Sturmberg and Martin 2013; Wenger 2010), cognitive theory (de Bruin and van Gog 2012; Flavell 1979; Schmidt et al. 1990) and social learning theories (Lave and Wenger 1991; Wertsch 1985). Theories of expertise development hold that learning to prescribe is a connected network of individual and social components including development of integrated knowledge, skills and understanding, activity within social contexts, response to feedback and self-reflection (Bereiter and Scardamalia 1993; McLellan et al. 2012; Moulton 2007). Given that individual and social aspects of learning were both highly relevant to our research question, a systemic view, that could take account of multiple aspects of learning, was more appropriate than privileging either the cognitive or social dimensions. Systems thinking allowed us to take inspiration from both of these discourses in educational theory, whilst preserving our view of learning to prescribe as a systemic whole. Insights from cognitive science heightened our awareness of the cognitive and metacognitive processes involved in learning to prescribe, while sociocultural perspectives directed our thoughts to the social environments and communities within which students exist. In simple systems, there are clear relationships between components, so cause and effect can be determined easily and outcomes can be predicted. Complicated systems are also predictable, but require detailed analysis and understanding of separate parts. Complex systems, in contrast, are unpredictable and the outcomes of interactions between their different elements can only be explained in retrospect. In complex systems, it is not possible to predict accurately how cause and effect will be linked, as aspects of the system are mutually interdependent. Whilst it is possible to determine key features of these systems and observe patterns of behaviour, their overall behaviour is emergent and evolves over time by a process of self-organisation (Snowden 2005). We were interested in whether our data were best explained by principles of simplicity, complicatedness or complexity.

### Methodology

This grounded theory research developed a theory inductively from data gathered using theoretical sampling, processes of data analysis and data collection, which informed each other, and constant comparative analysis. The grounded theory was constructivist (Charmaz 2006) in the sense that researchers constructed the theory by responding reflexively to what participants had said. Embracing Glaser’s maxim, “all is data” (Glaser 2001), we used data from multiple sources in order to establish a theory of how students learn to prescribe in the real world. A research team representing the perspectives of medical student (BN), Ph.D. student (LM), doctor (LM, SY, TD), educationalist (AdB, SY, TD), pharmacist (MPT) and cognitive scientist (AdB) worked to enhance each other’s reflexivity and challenge individual preconceptions. We did this by discussing and questioning each other’s individual analyses of the data, before agreeing on a final interpretation.

### Study context, sampling and recruitment

The study took place within the School of Medicine at the University of Manchester, which runs a problem-based curriculum. The first 2 years are predominantly non-clinical, although students have some early patient encounters. The next 2 years focus on the clinical application of principles learnt during years one and two. The final year of the programme is designed to prepare students to qualify as doctors by encouraging them to learn predominantly from real patients and integrate what they have learned in previous years. Although students have some basic science lectures relevant to prescribing within the pre-clinical years, the majority of undergraduate prescribing education takes place towards the end of the programme, so we selected final year medical students as our research participants. During their final year, students’ education occurs predominantly on clinical placement, rather than in lectures; therefore all participants were at the same stage in their training.

### Data collection and analysis

Data collection took place in three phases within a 27 months time frame, as required by the undergraduate timetable. During phase one, 10 participants recorded audio diaries for a period of 2 weeks, making entries whenever they encountered a situation which they felt was relevant to the development of their prescribing skills. As well as describing what happened, participants were invited to include their personal opinions and feelings about the situations they encountered.

Five of these participants participated in minimally structured qualitative interviews, in which LM asked them to reflect on specific experiences they had described in their audio diaries: the situation, nature of the learning experience, and reasons for making a recording about it. All diaries and interviews were transcribed verbatim and we completed an initial analysis of these data.

Phase two was observational, using direct observation and short in situ interviews. BN (a medical student on the fourth year of the same course) observed how learning occurred in workplaces and how hospital environments contributed to it. The observations, which totalled 42 h, took place over a 2-week period. Fourteen students were observed and ten of them gave interviews for qualitative analysis. BN did not actively participate in, or influence, the students’ activities and aimed to be as unobtrusive as possible throughout.

For phase three, we returned to our initial data collection methods but moved from purposive to theoretical sampling. We did this by seeking audio diary contributions from, and conducting interviews with students who had studied medical education and social learning theories. In line with our constructionist epistemology, we asked them to apply this perspective to their experiences of learning to prescribe and comment on some of the evolving themes within the existing data (see Table 1). We reasoned that their insights would be unique and valuable, due to their heightened awareness of social and contextual influences within their learning environments. We also recruited other students who we expected to give a more typical viewpoint and continued to collect data until no new themes were arising. At this point we agreed that we had reached theoretical saturation.

**Table 1:**
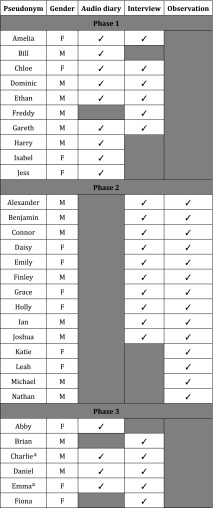
Details of participants

^a^Selected for participation due to their background in sociology and medical education

Participants for phases one and three were recruited by email. Phase two participants were recruited in situ by BN and, in keeping with our constructionist approach, were questioned about their understanding and interpretation of what BN had observed. All of them gave informed, written consent and were interviewed at times and locations convenient to themselves. The combination of audio diary, qualitative interview and observational research methods yielded a final data set comprising 20 interviews, 14 audio diaries and 2 weeks of observational field notes.

Data were analysed using a ‘template analysis’ approach, which is noted to be of particular value when studying real world settings (Brooks and King 2012) and is compatible with grounded theory methodology. LM produced a provisional coding template based on themes arising from a subset of data. This was revised multiple times, following discussions, as we collected more data and developed answers to the research question. Ultimately, we established themes which represented our complete data set and were able, by exploring the relationship between these themes, to generate a theory of how students learn to prescribe. All members of the research team contributed to developing the final interpretation.

Our findings are presented first as a report of the thematic data analysis and then as a conceptual model. This model was developed by iterating between relevant theory, outlined under the earlier heading ‘theoretical orientation’, and further analysing the empirical data. Where examples of the data have been shown, an ellipsis […] is used to indicate where words have been omitted. All participants have been given pseudonyms, which are appropriate to their gender and ethnicity. In explaining our interpretation, themes are highlighted in bold. Details of participants are shown in Table 1.

## Results

All participants talked about how individual and social factors influenced their experiences of prescribing and discussed the learning outcomes of various prescribing-related situations. Table 2 shows the coding template, categorized first into influencing factors and outcomes, then into more detailed descriptive themes. Given that these themes are so closely related and interlinked with one another, we have chosen not to substantiate each theme separately, but to present our theory and then use exemplars to describe their integrated nature.

**Table 2:** Coding template

First level code	Second level code	Third level code
Influencing factors	Individual learner	Metacognition
		Agency
		Motivation
		Identity
	Community	Social interaction
		Resources
		Role models
		Affordances
	Rules or boundaries	Legal restriction
		Curriculum
Outcomes	Experiential outcome	Teaching
		Learning
		Contribution to patient care
		Authenticity
	Pedagogical outcome	Knowledge building
		Whole-task practice
		Task understanding
		Work practices/strategies

### Theory of how students learn to prescribe

Many participants talked about their **motivations** for learning to prescribe, which included assessments, negative emotions (for example, fear and worry) and preparing for the upcoming responsibility of patient care. The extent to which participants were able to exercise **agency** (the ability to act autonomously) was, to a large extent, dependent on the **affordances** (properties that make action possible) of the social environments in which they learned. Many of them talked about being proactive on wards and seeking opportunities to meet personal learning goals. In order to do so, they often had to engage with the clinical community, either drawing on the expertise of others (often junior doctors and pharmacists), who they regarded as **role models**, or making use of **resources** such as drug charts and the drug formulary. Rules and boundaries influenced students’ learning; predominantly **legal constraints** and compulsory components of the **curriculum**.

Formal **teaching** sessions were often perceived to lack **authenticity** and, therefore, limit **learning**; however, some participants commented that teaching sessions still provided a valuable opportunity to build **knowledge** and the curriculum was viewed as having the capacity to bring prescribing into focus for both students and teachers. In workplaces, legal restrictions such as not being able to sign a drug chart presented a barrier to authentic opportunities to prescribe but sometimes these restrictions were negotiated so that a participant was afforded a chance to practice the task of prescribing in context. The workplace learning that participants described often failed to acknowledge the **whole-task** as a connected process, as experiences of integrating learning to prescribe with **contributing to patient care** were rare. Participants’ **understanding of the whole task** as an end-to-end process and their appreciation of the wider context of prescribing was limited.

Individuals began to position themselves as being competent as they aligned their own **identity** with that of a junior doctor and they talked about developing their own **work practices and strategies** for making the transition into working life. They did this by using **metacognition** to monitor and adapt their approaches to prescribing as they **interacted with the social world**. Conversely, while being able to project themselves into the role of prescriber for the purpose of reflection and self-regulation, students didn’t feel able to take on the identity of ‘prescriber’ fully, even under supervision, commenting that they could never become prepared for the task until they had true responsibility for it.

The following examples illustrate the theory described above and demonstrate the connected nature of the themes shown in our coding template.

*Example* 1

**Amelia (audio diary, phase 1):** “Since the final exams … I’ve been trying to actively look at what medications people are … on and then looking them up in the BNF (British National Formulary) to see why they’ve been prescribed, who they’ve been prescribed by, why the dosing regimen and … see if I can pick up any patterns, for example; Which medications are most cardiac patients on? What medications do you see in intensive care?…That’s been quite useful in that I’m starting to recognise the names and can put that to the actions of the different drugs.”

Self-**motivation** was the starting point for this prescribing experience. Amelia exercised **agency** in the workplace in order to engage with prescribing. She monitored her learning experience by reflecting on her cognitive process of pattern-recognition **(metacognition)**. As well as highlighting three of the individual factors contributing to learning, this example shows interaction with **resources** (the BNF) in the social world, the experiential outcome of **learning**, despite the absence of teaching, and the pedagogical outcome of **knowledge building**. This feeds back to **metacognition**, as she evaluated the outcome and perceived it as being useful to her learning.

*Example* 2

**Charlie (interview, phase 3):** “I guess the valuable knowledge was its mechanism of action, other uses, things like that, which wasn’t contextual at all, erm, yeah, we didn’t talk about why she [the patient] was started on it and when, so I still don’t understand that. Erm, and he [the doctor] asked a few questions. I answered them and then it stopped there, the message was almost ‘you know enough about this now.’ Well, actually, I don’t think I do.”

In this instance, Charlie described an inability to exercise **agency** due to a discrepancy between his own perception of what he needed to learn and the opinion held by his teacher (**social interaction**). He described an **inauthentic** approach to **teaching**, which didn’t **afford** him the opportunity to consider the **patient** despite the fact that this occurred within a workplace. Although contributing to **knowledge** building, this experience did not **afford** Charlie the opportunity to experience the **whole, end-to-end task** of prescribing (described in the introduction to this paper). He did, however, demonstrate **task understanding** in recognising the limitations of this opportunity. The problem caused by lack of integration between parts of the prescribing task and between prescribing and the rest of patient care, was summed up very well by one of our other participants:

**Dominic (interview, phase 1):** “I was left with my two, kind of, islands of knowledge about ‘what pneumonias are’ and ‘how to write a prescription’ and I couldn’t really link the two.”

In contrast, a different participant talked about the benefit of being able to experience the **whole task** whilst on GP placement. The workplace **afforded** her the opportunity to **contribute to patient care** by taking on responsibility for a consultation and experience the end to end process of meeting a patient, making a management decision, writing a prescription (which was subsequently checked and signed) and then following it up. Opportunities like this appeared to be rare:

**Emma (interview, phase 3):** “I actually had to learn to use the BNF, and I really needed to know which ones had penicillin and which didn’t and I really needed to know which bug was most likely to be causing it, and how to treat stuff…I actually was absolutely useless and it was embarassing…so, not only did it give me the motivation to learn that, but it also gave me the opportunity to prescribe it, get it signed by the GP and see the patient get better, or see him come back…I think that’s really one of the best learning opportunities I’ve had, because you got the continuity, you got the autonomy, you were left, you were out of your depth enough to struggle, flounder, but then… …you couldn’t do any harm.”

### Learning to prescribe: a complex adaptive social system

Links between participants’ interactions and the experiential and pedagogical outcomes, which ensued from them, were inconsistent. For example, it was noted during the observational phase of our study that students’ experiences of learning to prescribe during ward rounds were highly variable. The milieu was a social one in which interactions between students, patients, teachers, and other clinicians occurred in an inconsistent fashion despite the situation, from a curriculum perspective, apparently being consistent. These interactions were tacitly influenced by culture and social systems.

Our theory (presented above) provides evidence that outcomes of learning arose from socially situated, complex interactions. In view of the unpredictable nature of outcomes and the way in which they are sensitive to, but not controlled by, initial conditions, it may be useful to consider them emergent properties. In doing so, we propose that they cannot be explained by careful examination of individual elements of this system and that viewing learning to prescribe as a complex adaptive social system may provide helpful insights into influencing the system towards different emergent outcomes (Sweeney and Griffiths 2002). The utility of this view is that it allows acceptance of uncertainty, whilst still acknowledging the potential for influencing change.

Our data showed that learning to prescribe was embedded within the process of learning to be a doctor and prescribing itself was part of the bigger picture of caring for patients. Our model of a complex adaptive social system of learning to prescribe is situated at the intersection of two other overlapping systems: the medical curriculum and medical practice. That is to say that prescribing isn’t an independent, clearly defined component of learning to be a doctor or practising medicine, it is inextricably linked with everything else that is going on in the curriculum and in workplaces.

**Emma (audio diary, phase 3):** “Prescribing is nothing and everything, in some ways it isn’t a thing and in other ways it’s everything.”

**Fig. 1:**
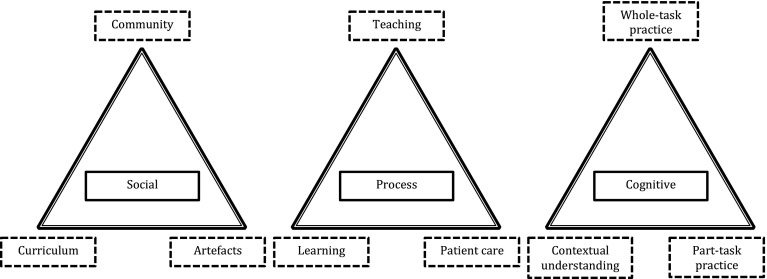
Three key dimensions of learning to prescribe: the ‘social space’, the ‘process space’ and the ‘cognitive space’. Students’ experiences of learning to prescribe can be positioned within each of these spaces, influenced by the variables at the *corners* of the *triangles*. The three dimensions are connected and influence each other. Due to the complexity of interactions, the system does not exhibit simple cause and effect relationships. For example, the same social conditions will not consistently achieve the same learning processes and cognitive outcomes

Characterising learning to prescribe using complexity as a conceptual framework, opens up the possibility of viewing a system made up of three important dimensions of the learning process; the social space, the process space and the cognitive space (Fig. 1). These represent layers of activity within a complex adaptive social system whose boundaries are flexible and permeable. Complexity is difficult to represent graphically because of its multidimensional nature but we have represented our findings as a pictorial model that may be of use to curriculum leaders and researchers who wish to explore the topic further:

We suggest that experiences of learning to prescribe are situated within these three ‘spaces’ simultaneously, as illustrated by the following example:

**Dominic (audio diary, phase 1):** “So, while we were in an out patients clinic I saw him [the resident] seeing a patient who had Bell’s palsy and he was going to prescribe him [the patient] a course of antivirals and steroids. At this point I thought well, you know, it would be quite good for me to take this opportunity to get my prescribing competency signed off, which he agreed to **(social space)**. However, it was a bit strange because I hadn’t really had a chance to speak to the patient and take a history, or do any examining, and he told me what to prescribe and what doses to prescribe as well **(process space)** so, at no point did I ever really interact with prescribing. I was more of a secretary taking dictation for him on the prescription sheet which he said he’d be willing to sign off, but it wasn’t at all like a real situation” **(cognitive space)**.

**Fig. 2:**
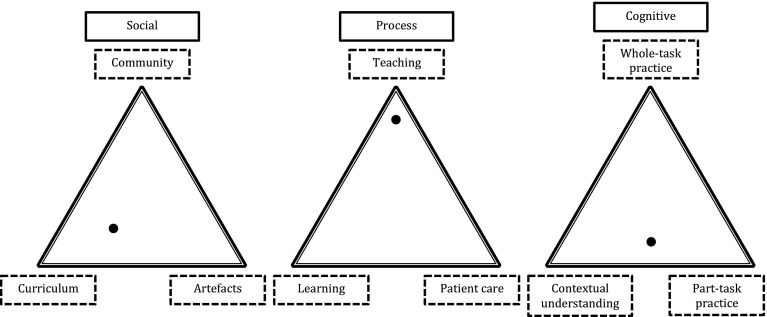
Worked example of the dimensions of learning to prescribe. The large *dots* are positioned according to our interpretation of where one participant’s experience (Dominic) of learning to prescribe would be located within these three dimensions. Future work could involve asking students to position their own experiences within this framework and collecting a large enough volume of data to be able to detect patterns of system behaviour

The first triangle of Fig. 2 shows that we positioned the social aspect of this experience as being located close to curriculum, but influenced by artefacts (the prescription sheet) and the community (the supervising doctor). The legal restriction of not being able to prescribe was negotiated, as the doctor allowed Dominic to participate in a way that he wouldn’t legally be able to take responsibility for. It was initiated by individual motivation to fulfil curriculum requirements and involved interaction with the resident in order to arrange this. In the process space, represented by the middle triangle of Fig. 2, we felt that this was mainly an example of teaching, as the student does not actively contribute to patient care and doesn’t indicate that much learning took place. Cognitively, this experience demonstrates part-task practice (filling in the paper work) and metacognition, as the student reflects on what has happened. This is illustrated in the third triangle of Fig. 2. An important feature of this illustration is that the triangles represent three dimensions of the same experience and are inseparable from one another. The position of any experience within these dimensions is a result of dynamic interactions and, accordingly, an imbalance in one space will have a direct effect upon the other two. For this reason, straightforward rules cannot be used to predict the positioning of future events. If Dominic were to attend the same outpatient clinic on a different day, the moment-by-moment decisions and interactions that took place would be likely to unfold differently, resulting in an experience positioned elsewhere within the spaces.

## Discussion

### Principal findings and meaning

We found that a complexity view of prescribing education, taking account of the cognitive and social aspects of learning, provided an informative conceptual framework and can offer insights that reductionist perspectives don’t afford. Our analysis suggests that students’ prescribing encounters are highly varied, due to the contexts in which clinical experiences occur. Although students were motivated to learn to prescribe, opportunities to practice the whole, contextualised task of prescribing in advance of becoming a qualified doctor were limited. It is, therefore, unsurprising that the transition from non-prescriber to prescriber is a significant challenge, potentially resulting in clinical errors.

In order to address our primary research question, we developed our thematic analysis into a model of how final year medical students learn to prescribe. This model illustrates a complex system, whose three major components are: the “social space”, where the environmental conditions influence or bring about a learning experience; the “process space”, describing what happens during the learning experience; and the intra-personal “cognitive space”, where the learner may develop aspects of prescribing expertise. Our thematic analysis established that the intricacy of interactions amongst individuals and between individuals and their social worlds made it hard to pinpoint straightforward ways of improving prescribing education, in which we could have any confidence. In order to analyse emergent patterns within these spaces, and develop interventions accordingly, a different methodological approach, using a larger data set, would be required. We anticipate that our model could provide a valuable framework for further work of this nature. The framework illustrates how cognitive and social aspects of learning can be viewed concurrently within a complex system perspective. We propose that it could be used as a reflective tool for students, who could reflect on the position of their experiences within the dimensions, and as an evaluation tool for teachers and researchers, who could identify connections between social aspects of learning and desirable or undesirable processes and cognitive outcomes.

### Relation to other publications

By conceptualising learning to prescribe as a complex system that bridges individualistic and socio-cultural learning theories, our work is in keeping with Billett’s notion that understanding workplace learning involves cultivating an appreciation of both the individual and the environmental and, crucially, the relationships between them (Harteis and Billett 2008a, b). We suggest that this also applies to learning that takes place outside workplaces, as we believe that formal components of the prescribing curriculum, although removed from authentic clinical contexts, can’t truly operate in isolation from the social realities of medical practice and workplaces. When we acknowledge the influence of both of these social spheres, education and practice, it becomes clear that viewing the system as a whole could yield benefits. Steven et al. studied factors that affect students’ learning in practice settings and demonstrated the importance of overlap between the practice of patient care and the practice of education. Viewing their model with systems thinking in mind, their work suggests that innovations encouraging overlap between the two practices could nudge the overall system towards favourable emergent properties (Steven et al. 2014). Bleakley confirms that viewing learning from a system perspective and “concentrating particularly upon the emergent properties of the system” opens up a “horizon of possibility” (Bleakley 2006) to view systems as dynamic wholes, rather than as the sums of their knowable parts. There is no shortage of literature applying complexity thinking to medicine and medical education (Fraser and Greenhalgh 2001; Mennin 2007, 2010; Plsek and Wilson 2001; Rees and Richards 2004; Sweeney and Griffiths 2002; Sweeney 2006), yet it remains relatively unfamiliar as an approach to medical education research (Bleakley 2010). We suggest that considering phenomena through a complexity lens provides a way of opening up assumptions of simplicity and reductionism, which the literature suggests are prominent in health care and medical education. That is not to say that existing theories are inadequate or should be abandoned but, simply, that an appreciation of complexity can help us to see the bigger picture. Ostensibly contradictory paradigms, such as cognitive science and sociocultural theories, are compatible within an overall complexity stance, as individual, social and environmental aspects of learning can be viewed as different layers, or dimensions, of an open and dynamic system (Bleakley 2010).

### Strengths and limitations

Throughout the study we carefully considered our theoretical assumptions, drawing on concepts from individualistic, social and system-based paradigms. This reflexive process was aided by the diversity of perspectives within our research team. We have placed our work upon firm theoretical foundations and the resulting theory is both grounded in the data and informed by established theory.

The time scale of our study meant that, on the one hand, we were able to capture perspectives across three different time periods, yet, on the other, we did not have sufficient data to be able to observe patterns of emergence. Additionally, we must appreciate that by investigating the system, we have become part of it, so our participants’ experiences may have been altered by their interaction with us. We don’t, however, feel that this reduces the validity of our findings, as the primary conclusion of this work is on a conceptual level. We are confident that the data are sufficient to justify our conclusions.

### Further work

Viewing learning to prescribe from a complex systems perspective means recognising that we can’t pre-determine all the effects of a single change as it could affect the way that many different components interact. Therefore, prior to introducing an educational intervention for undergraduate prescribing education, it is imperative to design an appropriate method of monitoring patterns of emergent learning experiences. We suggest that the three dimensions we have identified could provide a model for achieving this. Subsequently, adaptations to the system could be introduced on a trial and error basis and amplified or diminished depending on whether they have a positive or negative effect on students’ experiences of learning. Plsek and Greenhalgh (2001) support this method of observing patterns of emergence and intervening to gradually shift the system towards what appears to work best. To say that a phenomenon is complex is not to say that it presents an insurmountable challenge. It is entirely plausible that simple interventions may have beneficial effects on the emergent properties of this complex and multi-dimensional system.

## Conclusions

Learning to prescribe can be viewed as a complex adaptive social system, meaning that learning is viewed as occurring unpredictably, as a result of multiple, complex, socially situated interactions. We suggest that undergraduate prescribing research could be improved by increasing the potential for desirable learning opportunities to arise as emergent properties. By studying students’ experiences of learning to prescribe, we were able to develop a model outlining three different dimensions, or state spaces, within a complex system; social, process and cognitive. We present this model as a tool for further investigation of prescribing education, or workplace learning in general. By conceptualising this system and suggesting a framework for future research, we believe this work could provide an important foundation for improving the quality of undergraduate prescribing education and the safety of future prescribers. A complexity perspective calls for tolerance of ambiguity and an acceptance that not everything is knowable. This is not an excuse to surrender to a state of apathy but, instead, an invitation to rise to the challenge of exploring new ways of understanding the world.
